# Effect of Motility Factors D-Penicillamine, Hypotaurine and Epinephrine on the Performance of Spermatozoa from Five Hamster Species

**DOI:** 10.3390/biology11040526

**Published:** 2022-03-30

**Authors:** Maximiliano Tourmente, Ana Sanchez-Rodriguez, Eduardo R. S. Roldan

**Affiliations:** 1 Department of Biodiversity and Evolutionary Biology, Museo Nacional de Ciencias Naturales, Spanish Research Council (CSIC), 28006 Madrid, Spain; anasanro.vet@gmail.com; 2 Centro de Biología Celular y Molecular, Facultad de Ciencias Exactas, Físicas y Naturales, Universidad Nacional de Córdoba, Cordoba X5016GCA, Argentina; 3 Instituto de Investigaciones Biológicas y Tecnológicas (IIByT), Consejo Nacional de Investigaciones Científica y Técnicas (CONICET), Cordoba X5016GCA, Argentina

**Keywords:** sperm performance, hamster, sperm motility, acrosome integrity, ATP content, *Mesocricetus auratus*, *Cricetulus griseus*, *Phodopus sungorus*, *Phodopus roborovskii*, *Phodopus campbelli*

## Abstract

**Simple Summary:**

Analysis of sperm performance under in vitro conditions provides a good indication of fertilizing potential. Parameters such as motility, swimming kinetics, acrosome integrity, or ATP content are thus examined in efforts to characterize such potential. Hamster species are a good model to study sperm parameters that are key determinants of fertilizing capacity because these species are at the higher end of the diversity of mammalian sperm morphology and performance. In vitro functional studies demand that sperm remain viable during a long period of time under conditions that resemble those in the female tract. Sperm from certain species require supplementation of the incubation medium with factors that stimulate viability and swimming, or that promote acquisition of fertilizing capacity. Molecules important for sperm performance in hamsters have been identified, namely D-penicillamine, hypotaurine and epinephrine (PHE). In the present study, we investigated the effect of PHE on spermatozoa from five hamster species incubated for up to 4 h. Our results revealed that PHE maintains sperm performance in the golden hamster, whereas it improves sperm quality in the Chinese hamster. In contrast, it does not seem to have any effect on sperm from the Siberian (Djungarian), Roborovski and Campbell’s dwarf hamsters. These results are valuable to understand the different regulatory mechanisms of sperm motility and survival in different species.

**Abstract:**

Assessments of sperm performance are valuable tools for the analysis of sperm fertilizing potential and to understand determinants of male fertility. Hamster species constitute important animal models because they produce sperm cells in high quantities and of high quality. Sexual selection over evolutionary time in these species seems to have resulted in the largest mammalian spermatozoa, and high swimming and bioenergetic performances. Earlier studies showed that golden hamster sperm requires motility factors such as D-penicillamine, hypotaurine and epinephrine (PHE) to sustain survival over time, but it is unknown how they affect swimming kinetics or ATP levels and if other hamster species also require them. The objective of the present study was to examine the effect of PHE on spermatozoa of five hamster species (*Mesocricetus auratus*, *Cricetulus griseus*, *Phodopus campbelli*, *P. sungorus*, *P. roborovskii*). In sperm incubated for up to 4 h without or with PHE, we assessed motility, viability, acrosome integrity, sperm velocity and trajectory, and ATP content. The results showed differences in the effect of PHE among species. They had a significant positive effect on the maintenance of sperm quality in *M. auratus* and *C. griseus*, whereas there was no consistent effect on spermatozoa of the *Phodopus* species. Differences between species may be the result of varying underlying regulatory mechanisms of sperm performance and may be important to understand how they relate to successful fertilization.

## 1. Introduction

Sperm performance, a major determinant of male fertility, can be dissected into a series of traits that are intricately connected to sperm fertilizing potential [1,2]. Therefore, a combination of various performance parameters is usually assessed to evaluate the quality of sperm samples and predict its fertilizing potential in man and other animals [2,3,4,5,6,7,8,9,10]. Sperm viability, motility and kinetics, as well as acrosome integrity, are all linked to sperm survival. The proportion of motile sperm and the velocity of spermatozoa are essential for sperm to swim along the cervix, uterus and the uterotubal junction, and cells that show higher values in these parameters also have higher chances to reach and fertilize the ovum [11]. The assessment of sperm motility may be carried out subjectively, estimating the percentage of motile sperm by microscopic visualization, and objectively through the quantification of sperm swimming parameters by computer aided sperm analysis (CASA) [1,2,10,12,13].

The sperm acrosome contains enzymes that are released during exocytosis, an essential step required for penetration of the oocyte’s vestments [14]. The timing of release is important, so the integrity of the acrosome during sperm transport in the female tract and, in particular, along the oviduct, has to be preserved in order to ensure fertilizing potential [15]. Controversy still exists as to which is the site where acrosomal exocytosis takes place or which is the physiological ligand (or ligands) responsible for initiating exocytosis [16,17,18,19]. Nevertheless, since this is a key event in the processes leading to completion of fertilization, acrosomal status has become a valuable assessment of fertilizing potential [20].

Other relevant sperm parameters relate to sperm bioenergetics, with sperm ATP content serving as an indicator of the balance between sperm ATP production and consumption [21]. High ATP levels are positively correlated to sperm swimming velocity in rodents [22] and mammals in general [23]. Moreover, assessments of the variation in sperm ATP content and sperm traits over time among rodent species revealed that maintenance of high performance in species with high competitive ability is associated with high concentrations of intracellular ATP over time [22,24,25].

Exogenous factors could influence sperm performance in vivo and under in vitro conditions [26]. Besides the provision of energetic substrates that are fundamental for the production of ATP for sperm motility and survival, conditions in the female tract promote the acquisition of sperm’s fertilizing ability, a process known as capacitation [27]. Among physical factors, extracellular pH, temperature, and viscosity are known to affect the survival and performance of sperm cells [28,29,30,31,32,33,34,35]. Several biological factors are also known to have important roles in sperm survival in vivo and in vitro. Early efforts to achieve in vitro fertilization had to rely on homologous or heterologous serum to ensure sperm survival and the acquisition of fertilizing ability [36,37]. Better definition of media was possible with replacement of serum by bovine serum albumin. However, these media were still not completely defined [38]. Components of the extracellular milieu may be required to sustain sperm motility, and variations between species may exist regarding the nature of such components. For golden hamster (*Mesocricetus auratus*) spermatozoa, one set of important motility factors are catecholamines, which maintain and stimulate sperm motility in vitro [39]. Within the group of catecholamines, epinephrine is an essential co-factor for golden hamster sperm as it activates motility as well as Na^+^/K^+^ ATPase and Ca^2+^-ATPase [40], and it is also involved in the acquisition of fertilizing ability [41]. However, epinephrine is not able to maintain golden hamster sperm motility on its own, and a second factor, hypotaurine, is also essential. Hypotaurine is a superoxide scavenger that functions inhibiting lipid peroxidation and superoxide dismutase [42], which prevents motility loss. Hypotaurine and epinephrine together also cause a mild increase of the acrosome reaction in hamster sperm [43]. Other factors have been shown to influence hamster sperm function [44,45,46].

Another molecule of interest, with regards to hamster sperm survival, is D-penicillamine. This is an α-amino acid which acts as a cation chelator, protecting sperm from oxidation in several species [40,47,48,49,50]. As a zinc chelator, D-penicillamine facilitates capacitation, acting in the early step of this process because it removes most of the zinc within the first ten minutes of its addition, but it is not enough to support full capacitation [51]. D-penicillamine also prolongs hyperactivated motility [52]. This amino acid together with epinephrine and hypotaurine (PHE: penicillamine + hypotaurine + epinephrine) act as motility factors, necessary for the maintenance of golden hamster sperm motility in vitro [51]. The addition of PHE to incubation media maintains golden hamster sperm motility within the first hour [53,54] and also promotes sperm capacitation in this [54] and other species [55]. The synergistic effect of the three components of PHE can reactivate immotile spermatozoa of golden hamsters [56].

Many early studies of sperm function have used the golden hamster as a model, particularly because of the ease of examining acrosomal status in motile spermatozoa [57]. Studies of sperm behavior in a related species, the Chinese hamster (*Cricetulus griseus*), have been performed [58,59,60] and these studies have also identified the need to support sperm viability and motility over time to achieve fertilization. Similar requirements seem to exist for Siberian hamster (*Phodopus sungorus*) spermatozoa because low success was achieved in in vitro fertilization with a variety of media and supplements [61], but characterization of these requirements has not been undertaken. Hamsters are a valuable model for studies of sperm-oocyte recognition and interaction. Hybridization has been observed among hamsters (*Mesocricetus* species: [62,63,64,65,66]; *Phodopus* species: [67]; *Allocricetulus* species: [68]), and cross-fertilization in vitro between golden and Chinese hamsters has been reported [37]. Despite the potential of this group of species as a model for sperm biology, little is still known about the spermatozoa and fertilization of most species in this group. Comparative and evolutionary studies are needed to understand diversity in function and underlying mechanisms of sperm survival, capacitation and acrosomal exocytosis. Hamster species are attractive because they are at the higher end of the range of mammalian sperm dimensions [69,70], have high sperm swimming velocities [22] and exhibit high levels of sperm ATP [22,24]. Such traits, characterizing these species as high performers, may be the result of intense sperm competition [71]. Detailed comparative studies of sperm performance may therefore be rewarding in hamsters of the subfamily *Cricetinae* for which good background information of phylogenetic relations currently exists based on molecular studies [72] and chromosome evolution [73,74].

In the present study, we evaluated whether several biological molecules had a role in the performance of spermatozoa from five different species of hamster: *Mesocricetus auratus*, *Cricetulus griseus*, *Phodopus campbelli*, *P. sungorus* and *P. roborovskii*, focusing on sperm motility, viability, sperm kinematics, acrosome integrity, and bioenergetics. Spermatozoa from these species are at the higher end of the range of sperm quality parameters among rodent species [22,24]; thus, a detailed analysis of modulators of sperm function in these species could help understand determinants of male fertility.

## 2. Materials and Methods

### 2.1. Reagents

Unless stated otherwise, reagents were purchased from Sigma or Merck (both of Madrid, Spain).

### 2.2. Animals and Sperm Collection

Adult males (4–6 months old) of *Cricetulus griseus* (n = 5), *Mesocricetus auratus* (n = 6), *Phodopus campbelli* (n = 5), *P. sungorus* (n = 7), and *P. roborovskii* (n = 5) were kept in captivity in our animal facilities. Animals were maintained under standard conditions (14 h light–10 h darkness, 22–24 °C), with food and water provided ad libitum. Each male to be used in this study was housed alone (i.e., in individual cages) for at least one month before sperm collection. Males were sacrificed by cervical dislocation and weighed immediately. Testes were then removed and weighed. Relative testes size was calculated using the potential equation defined for rodents by Kenagy and Trombulak [75].

The caudae epididymides were excised after removing all blood vessels, fat, and surrounding connective tissues. Each cauda epididymis was placed in a Petri dish containing one of two variants of culture medium pre-warmed to 37 °C and spermatozoa were collected by performing three to five incisions in the distal region of the cauda, and allowing them to swim out for 5 min. As standard medium (“control” treatment), we used a Hepes-buffered modified Tyrode medium with albumin (see mT-H in [76]), with the addition of lactate and pyruvate (mTALP: 120.89 mM NaCl, 2.68 mM KCl, 0.49 mM MgCl_2_·6H_2_O, 0.36 mM NaH_2_PO_4_·2H_2_O, 20 mM Hepes, 1.80 mM CaCl_2_, 5.56 mM glucose, 1 mM sodium pyruvate, 10 mM sodium lactate, 4 mg mL^−1^ bovine serum albumin) under air (pH 7.4). A modified medium (PHE treatment) was mTALP supplemented with 20 µM D-penicillamine, 100 µM hypotaurine, and 1 µM epinephrine. Sperm suspensions were placed in plastic tubes, where sperm concentration was estimated by using a modified Neubauer chamber and adjusted to 20 × 10^6^ sperm mL^−1^ by diluting with medium. Sperm parameters were assessed in the sperm suspensions corresponding to each treatment immediately after adjusting the concentration (hereafter referred as 0 h), and after 2, 3, and 4 h of incubation at 37 °C under air. Large-bore pipette tips were used to minimize damage to spermatozoa in all procedures.

### 2.3. Sperm Motility, Viability and Acrosomal Integrity

Sperm motility was evaluated by examining 10 µL of a previously diluted sperm suspension, placed between a pre-warmed slide and a coverslip, at 100× magnification under phase-contrast optics. The percentage of motile sperm was estimated by at least two independent, experienced observers, whose estimations were averaged and rounded to the nearest 5% value.

The assessment of sperm viability and acrosome integrity was performed by staining sperm first with eosin-nigrosin and subsequently with Giemsa [77]. Briefly, 5 µL sperm suspension and 10 µL eosin-nigrosin solution were mixed on a glass slide placed on a stage at 37 °C and 30 s later the mix was smeared and allowed to air-dry. Smears were fixed by immersion during 10 min in a solution of 4% formaldehyde in TPB buffer. After fixation, the smears were stained with Giemsa solution and mounted with DPX. Slides were examined at 1000× under bright field and 200 spermatozoa per male were examined to evaluate sperm viability and integrity of the acrosome. Viable spermatozoa were those excluding eosin (from the eosin-nigrosin stain). Acrosome integrity was reported as the percentage of sperm with intact acrosomes, excluding cells that showed damaged or missing acrosomes.

### 2.4. Sperm Velocity and Trajectory

To assess sperm swimming velocity and trajectory, an aliquot of sperm suspension was placed in a pre-warmed microscopy chamber with a depth of 20 µm (Leja, Nieuw-Vennep, The Netherlands) and filmed using a phase contrast microscope with pseudo-negative phase connected to a digital video camera (Basler A312fc, Vision Technologies, Glen Burnie, MD, USA). A 4× objective was used instead of the 10× objective traditionally used for CASA analysis on sperm from humans and domestic animals. This resulted in larger field of observation, which allowed for tracking of the unusually large and fast hamster sperm for longer, and a deeper focal plane to account for the depth of the observation chamber. Sperm trajectories were assessed using a computer aided sperm analyzer (Sperm Class Analyzer—SCA v.4.0, Microptic, Barcelona, Spain), and the following swimming parameters were estimated for each track: curvilinear velocity (VCL, µm s^−1^), straight-line velocity (VSL, µm s^−1^), average path velocity (VAP, µm s^−1^), linearity (LIN = VSL/VCL), straightness (STR = VSL/VAP), wobble (WOB = VAP/VCL), amplitude of lateral head displacement (ALH, µm), and beat-cross frequency (BCF, Hz). The software was set with frame rate 50 s^−1^, maximum particle size 500 μm, minimum particle size 50 μm, connectivity 30, contrast 400, and brightness 160. All video captures were compared to their overlaying analyzed tracks, and trajectories that did not belong to sperm were removed. In addition, trajectories with VAP values lower than 20 µm s^−1^, and LIN, STR and WOB values of 100 in the post-capture analysis were discarded as these are typical of drifting but immotile sperm or other non-sperm particles. Since spermatozoa of *Cricetulus griseus* are significantly larger than those of the other species studied (about 250 µm [69]), and their head has a slender falciform shape that makes it almost undistinguishable from the midpiece in the moving sperm, the SCA software was not able to obtain accurate sperm trajectories. Thus, data on sperm velocity and trajectory for *C. griseus* are not presented.

### 2.5. Sperm ATP Content

Sperm ATP content was measured using a luciferase-based ATP bioluminescent assay kit (Roche, ATP Bioluminescence Assay Kit HS II), as previously described [22,24]. A 100 µL-aliquot of diluted sperm suspension was mixed with 100 µL of Cell Lysis Reagent, vortexed and incubated at room temperature for 5 min. The resulting cell lysate was centrifuged at 12,000× *g* for 2 min, and the supernatant was recovered and frozen in liquid N_2_. Bioluminescence was measured in triplicate in 96-well plates using a luminometer (Varioskan Flash, Thermo Fisher Scientific Inc., Waltham, MA, USA). In each well, 50 µL of Luciferase reagent were added to 50 µL of sample (via auto-injection), and, following a 1 s delay, light emission was measured over a 10 s integration period. Standard curves were constructed using solutions containing known concentrations of ATP diluted in mTALP and Cell Lysis Reagent in a proportion equivalent to that of the samples. ATP content was expressed as amol sperm^−1^.

### 2.6. Data Analysis

#### 2.6.1. Principal Component Analyses for Sperm Velocity Parameters

**Figure 1:**
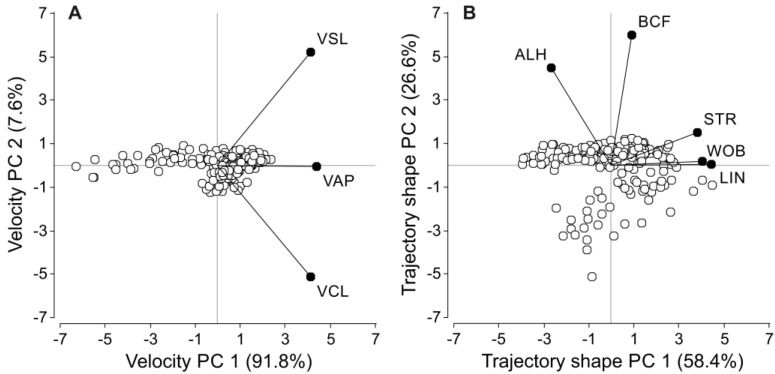
Biplots detailing the relationships between principal components of sperm velocity (**A**) and trajectory shape (**B**) and their constituent variables. Variable values were log_10_ or arcsine transformed prior to analysis. VCL: curvilinear velocity. VSL: straight-line velocity. VAP: average path velocity. LIN: linearity. STR: straightness. WOB: wobble coefficient. ALH: amplitude of the lateral head displacement. BCF: beat-cross frequency.

Since swimming parameters tend to be highly correlated [78], principal component analyses (PCA) were performed to construct variables that summarize the information obtained through CASA. The variables were divided in two groups defining sperm velocity (VCL, VSL, VAP) and sperm trajectory (LIN, STR, WOB, ALH, BCF) and one independent PCA was carried out for each group. The first principal component for the velocity group (VPC1) accounted for 91.8% of the variability in the three summarized variables (VCL, VSL, VAP), while the second principal component (VPC2) only accounted for 7.6% of the variability (Figure 1A). The three sperm velocity descriptors showed extremely high correlation coefficients with VPC1 and were weakly or non-significantly correlated with VPC2 (Table 1). For the trajectory, the first principal component (TPC1) accounted for 58.4% of the variability, while the second principal component (TPC2) represented 26.6% of the variability (Figure 1B). Although the five variables included in this group were correlated with TPC1, the strength of the correlation was higher for LIN, STR, and WOB (Table 1). On the other hand, ALH and BCF showed a stronger correlation with TPC2 (Table 1). Thus, VPC1, TPC1 and TPC2 values for each treatment and species were used as our integrated sperm velocity and trach shape measures.

#### 2.6.2. Statistical Analyses

The effect of incubation with PHE on sperm parameters over time was analyzed with a two-factor repeated-measures ANOVA for each species, using treatment (2 levels: control and PHE) and time (4 levels: 0, 2, 3, 4 h) as factors. Differences between conditions were analyzed through a post-hoc DGC multiple comparisons test [79]. All variables were log_10_-transformed for statistical purposes, with the exception of percentages (motility, viability, acrosome integrity, LIN, STR and WOB), which were arcsine-transformed. The statistical analyses were performed using SPSS Statistics (SPSS v.23.0.0.0; SPSS, IBM Corporation, Somers, NY, USA), and InfoStat v.2015p (Grupo Infostat, Universidad Nacional de Córdoba, Córdoba, Argentina) with α = 0.05.

## 3. Results

### 3.1. Relative Testes Size and Sperm Numbers

Body mass, testes mass, relative testes size and sperm numbers per individual are presented in Table 2. The mass of testes in relation to body mass was high in all species when compared with other species of muroid rodents (see [22,77]). Among the species examined here, *Cricetulus griseus* showed the highest relative testes size (*sensu* [75]), while *Phodopus sungorus* presented the smallest testes in relation to body mass.

### 3.2. Sperm Motility, Viability, Acrosome Integrity and ATP Content

**Figure 2:**
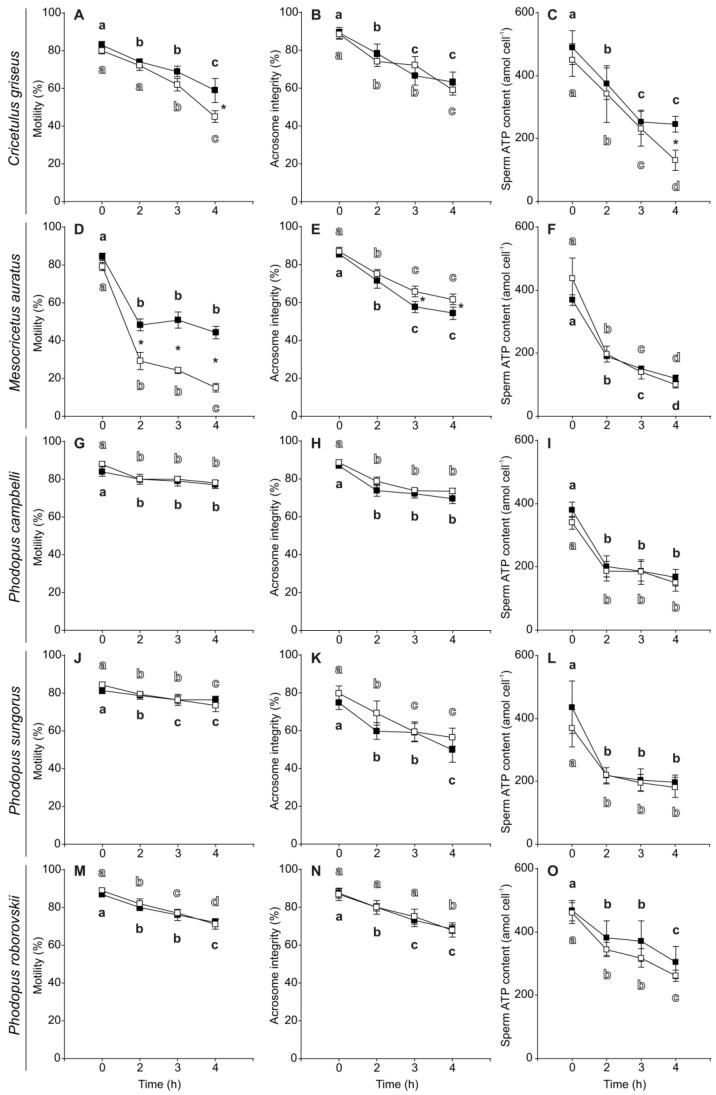
Changes in motility, acrosome integrity, and ATP content in spermatozoa from five hamster species during incubation without or with penicillamine, hypotaurine and epinephrine (PHE). Spermatozoa were collected in medium with (black squares) or without PHE (white squares) and incubated at 37 °C under air for up to 4 h. (**A**,**D**,**G**,**J**,**M**) Percentage of motile sperm. (**B**,**E**,**H**,**K**,**N**) Percentage of spermatozoa with an intact acrosome. (**C**,**F**,**I**,**L**,**O**) Sperm ATP content (amol × cell^−1^). Values are means ± standard errors. Different letters between times of incubation for the same treatment indicate statistically significant differences (*p* < 0.05) in a DGC post-hoc test. Asterisks indicate statistical differences (*p* < 0.05) between treatments for the same time in a DGC post-hoc test.

Initial sperm motility (~80–90%), viability (~95–99%), and acrosome integrity (~75–90%) were high for all species in both media (Table 3). In addition, sperm of the five species showed relatively high initial ATP content in media without and with PHE (Table 3) when compared with other muroid rodent species (see [24,80]). In general, sperm showed a significant gradual decline in their motility and acrosome integrity throughout the 4 h of incubation (Table 4, Figure 2) with the exception of *Mesocricetus auratus* spermatozoa which exhibited a more pronounced decrease in motility between 0 and 2 h of incubation, with a slight decrease afterwards (Figure 2D). Sperm ATP content also decreased significantly with time in the five species (Table 4), with a pronounced phase of decline between 0 and 2 h of incubation in four of the five species (Figure 2), and a more constant decrease over the 4 h of incubation in the case of *C. griseus* (Figure 2C). Sperm viability did not change significantly during incubation time in any of the five species, maintaining high values (>90%) in all species with both treatments (Table 4, Figure S1).

The incubation in a medium with PHE showed a significantly positive effect on sperm motility in *C. griseus* and *M. auratus* (Table 4). The effect was stronger and appeared earlier in *M. auratus* (2 h of incubation, Figure 2D) than in *C. griseus* (4 h of incubation, Figure 2A). In *C. griseus*, sperm viability was significantly higher in the PHE treatment than in the control after 2 h of incubation (Table 4, Figure S1A). Conversely, *M. auratus* sperm showed differences favoring the control over the PHE treatment at 3 and 4 h (Table 4, Figure S1B). In the case of *Phodopus* species, the addition of PHE to the incubation medium showed no significant effects on sperm motility, viability, and acrosome integrity (Table 4, Figure 2 and Figure S1). Sperm ATP content was not affected by the addition of PHE to the incubation medium (Table 4, Figure 2) with the exception of *C. griseus* in which PHE promoted a less pronounced decline in ATP content (Table 4, Figure 2C).

### 3.3. Sperm Velocity and Trajectory

**Figure 3:**
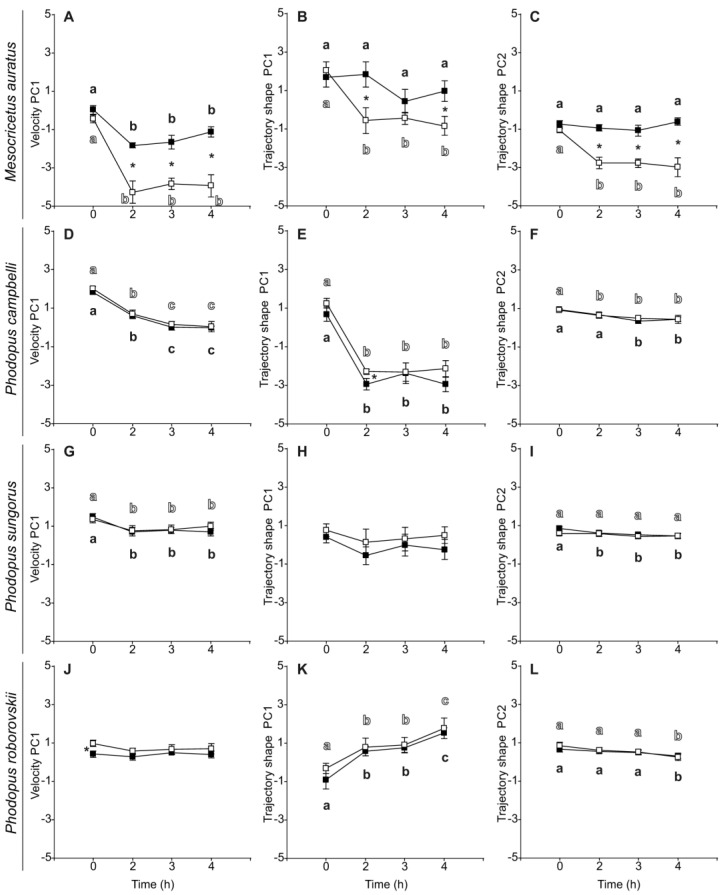
Changes in velocity or trajectory of spermatozoa from five hamster species during incubation in the absence (control) or the presence of penicillamine, hypotaurine and epinephrine (PHE). Spermatozoa were collected in medium with (dark squares) or without PHE (white squares) and incubated at 37 °C under air for up to 4 h. Principal component analysis returned one main velocity component (velocity PC1) and two trajectory components (trajectory shape PC1, trajectory shape PC2). (**A**,**D**,**G**,**J**) Velocity PC1. (**B**,**E**,**H**,**K**) Trajectory shape PC1. (**C**,**F**,**I**,**L**) Trajectory shape PC2. Values are means ± standard errors. Different letters between times of incubation for the same treatment indicate statistically significant differences (*p* < 0.05) in a DGC post-hoc test. Asterisks indicate statistical differences (*p* < 0.05) between treatments for the same time in a DGC post-hoc test.

Regarding sperm velocity and trajectory parameters, all species presented similar values regardless of treatment at the beginning of the incubation period (0 h) (Table 5). As a general trend, velocity and trajectory parameters, as well as their estimated principal components (VPC1, TPC1, and TPC2) exhibited a time-related decline in both control and PHE treatment (Table 6, Figure 3). However, several variables did not show this time-related effect in some species. The sperm of *M. auratus* that were incubated in the presence of PHE maintained their linearity, straightness, and amplitude of lateral head displacement throughout the incubation period (Table 6, Figure 3B,C). In *P. sungorus*, straightness, amplitude of lateral head displacement, beat-cross frequency, and TPC1 were similar during the 4 h incubation regardless of treatment (Table 6, Figure 3H). This pattern was also observed in the values of linearity, and TPC2 but only for the control treatment (Table 6, Figure 3I). In *P. roborovskii*, sperm straight-line velocity, average path velocity, and VPC1 did not decrease over time (Table 6, Figure 3J). Notably, in this species, sperm linearity, straightness, wobble coefficient, and TPC1 registered a gradual increase during incubation time (Table 6, Figure 3K).

The sperm of *M. auratus* exhibited a strong significant response to the exposure to PHE during the incubation, in regard to their velocity and trajectory parameters. All velocity and trajectory variables, including their estimated principal components, showed significant higher values in the PHE treatment (Table 6, Figure 3A–C). The addition of PHE to the medium appears to prevent the time-related decline of sperm kinetics in this species. Conversely, the addition of PHE did not appear to have any positive effect on the kinetics of the sperm of any of the three species of the genus *Phodopus*. As a general trend, there were no significant differences in the values of most of the sperm velocity and trajectory variables in *Phodopus* species (Table 6, Figure 3D,F–I,K,L). In the few cases in which significant differences were observed (*P. campbelli*: VSL, LIN, WOB, ALH, and TPC1; *P. sungorus*: LIN, WOB; *P. roborovskii*: VCL, VSL, VAP, WOB, VPC1), these differences were of relatively low magnitude, were not maintained over time, and values were always higher in the control treatment (Table 6, Figure 3E,J).

## 4. Discussion

The results of this study show that the addition of D-penicillamine, hypotaurine and epinephrine (PHE) to the incubation medium seems to be necessary to sustain the performance (motility, sperm swimming velocity and trajectory) and, to a lesser extent, acrosome integrity and viability, of *M. auratus* sperm throughout incubation. Moreover, PHE appears to have a positive effect on the maintenance of sperm quality (motility, viability, and ATP content) in *C. griseus*. In contrast, the presence of PHE had no consistent effect on spermatozoa of the *Phodopus* species.

Golden hamster sperm is known to be very susceptible to in vitro dilution for more than 15–30 min [81,82] unless the medium is supplemented with motility factors. The addition of epinephrine, taurine, hypotaurine, penicillamine, or their combination, to hamster sperm has been studied mainly in *M. auratus* [39,40,56,81,82,83,84,85]. In the present work, a combination of D-penicillamine (20 µM), hypotaurine (100 µM) and epinephrine (1 µM) was selected to analyze their effects on sperm performance. Our results showed an improvement in motility, integrity of the acrosome and velocity and trajectory parameters from 2 h onwards after addition of PHE in *M. auratus*. Several studies showed that epinephrine and hypotaurine have a synergistic effect in maintaining sperm motility and promoting the acrosome reaction [40,43]. These two molecules caused a slight enhancement in motility, acrosome reaction and fertility when added separately, and these effects were increased when used together [83]. Moreover, the effect of hypotaurine occurs within the first 2 h [83], in agreement with the present study, where sperm motility, velocity and trajectory showed better results 2 h after the addition of PHE in *M. auratus*.

Epinephrine is a catecholamine that stimulates Na^+^/K^+^ ATPase and Ca^2+^-ATPase, and inhibits certain phosphodiesterases [40,86,87]. The inhibition of Na^+^/K^+^ ATPase decreased the acrosome reaction, whereas the inhibition of phosphodiesterase increased it [85]. Hypotaurine is a β-amino acid present in ejaculated sperm and oviductal fluid of mammals [43]. It is an intracellular scavenger that protects from lipid peroxidation and inactivation of superoxide dismutase by superoxidation, preventing sperm motility loss [84]. D-penicillamine acts as a divalent cation chelator, increasing the effects of epinephrine by protecting it from oxidation [40]. It was found that this α-amino acid could maintain sperm motility in hamsters at lower concentrations (10 µM) and stimulate sperm capacitation at higher concentrations (50 µM) [51]. In the present study, the concentration of penicillamine was 20 µM, which seems to be adequate in *M. auratus* to maintain or improve sperm traits for 4 h, in combination with epinephrine and hypotaurine. Taken together, results lead to the conclusion that the addition of PHE to *M. auratus* spermatozoa improves sperm quality over time, probably through the interaction with ATPases and phosphodiesterases and by protecting sperm from oxidation.

Changes in sperm traits in *C. griseus* after the addition of PHE are different from those observed in *M. auratus*. In *C. griseus* sperm motility and ATP content are enhanced at a later time, i.e., after 4 h of incubation with PHE. Moreover, sperm viability improves from 2 h onwards. In an earlier study [58], it was found that only 20–30% of cauda epididymal *C. griseus* sperm exhibit motility, which is significantly lower than the results in our study. This discrepancy may be due to differences in media used to collect and incubate spermatozoa because in the earlier study [58] the medium contained epinephrine and taurine (instead of hypotaurine, as in our study), and lacked penicillamine. The improvement in sperm survival and performance observed in our work may be useful to improve the reduced in vitro fertilization success observed in this species [58,88]. Indeed, better fertilization rates were obtained when sperm were pre-exposed to PHE and to the Ca^2+^ ionophore A23187, while doubling the usual Ca^2+^ concentrations [60].

Different patterns of sperm motility have been found when comparing *M. auratus* with *C. griseus* [58]. Whereas *M. auratus* sperm flex the entire length of the tail when moving, *C. griseus* sperm move by vibrating the tails firmly. This difference in swimming patterns may explain the diverse results in both species. Despite these differences, the addition of PHE seems to promote an improvement of sperm performance in both species. There does not seem to be a relation between the presence or absence of PHE, ATP levels, and motility when comparing these two species. In *M. auratus* PHE sustains motility better and for longer times than in controls, but there does not seem to be an effect on ATP levels. In *C. griseus*, there seems to be a parallel improvement of PHE on both motility and ATP levels, but only at the end of incubation.

The results in *Phodopus* species are somewhat surprising because we did not find differences over time in variables assessed in sperm incubated in the absence or presence of PHE. To the best of our knowledge, this is the first report examining the effects of PHE in these three species of hamsters. Since previous studies found that epinephrine acts through α and β-adrenergic receptors [41] and hypotaurine activates ATPases and inhibits phosphodiesterases, differences between sperm from *Phodopus*, *C. griseus* and *M. auratus* may be explained by differences in receptors or signaling mechanisms. Thus, one possibility could be that targets of PHE action vary in concentration between species, and thus effects are not visible. Another possibility is that responses to each factor vary, since different concentrations of penicillamine, hypotaurine and epinephrine are known to have different effects in somatic and sperm cells [51,86,87]. More studies are necessary to elucidate if changes in concentrations of PHE may affect sperm traits in *Phodopus* species. In any case, sperm quality in these hamsters is extremely high when compared to other hamster species after 4 h of incubation without PHE, indicating that the addition of these molecules or other motility factors may not be required to sustain sperm performance.

## 5. Conclusions

The results of this study revealed that supplementation with a combination of D-penicillamine, hypotaurine and epinephrine maintains or improves the performance of spermatozoa from five hamster species in different manners, depending on the species. Further studies are needed to understand the different mechanisms underlying the stimulatory effects in some species and lack of stimulation in others, which suggest divergence in regulatory mechanisms of motility and survival.

## Figures and Tables

**Table 1:** Loadings and correlation of sperm traits with principal components of sperm velocity and trajectory shape in five hamster species. Values presented are Pearson’s correlation coefficients. Significant correlation coefficients (*p* < 0.05) are shown in bold. PC1: principal component 1. PC2: principal component 2. Variable values were log_10_ or arcsine transformed prior to analysis.

Variables	Factor Loadings		Factor Correlation	
	PC1	PC2	PC1	PC2
Sperm velocity principal components:				
Curvilinear velocity	0.5667	−0.7023	**0.9405**	**−0.3345**
Straight-line velocity	0.5658	0.7118	**0.9390**	0.3390
Average path velocity	0.5989	−0.0079	**0.9938**	−0.0038
Sperm trajectory shape principal components				
Linearity	0.5800	0.0041	**0.9914**	0.0047
Straightness	0.5008	0.1996	**0.8560**	0.2302
Wobble coefficient	0.5261	0.0217	**0.8992**	0.0251
Amplitude of lateral head displacement	−0.3498	0.5869	**−0.5980**	**0.6767**
Beat-cross frequency	0.1167	0.7844	**0.1995**	**0.9045**

**Table 2:** Corporal measurements and sperm numbers in five hamster species. Values indicate mean ± standard error. RTS: relative testes size calculated according to Kenagy and Trombulak [75].

Species	Body Mass (g)	Testes Mass (g)	RTS	Sperm Numbers (×10^6^)
*Cricetulus griseus*	33.72 ± 0.38	1.78 ± 0.04	3.83 ± 0.11	88.00 ± 07.65
*Mesocricetus auratus*	125.00 ± 1.63	3.50 ± 0.12	2.75 ± 0.11	585.88 ± 36.81
*Phodopus campbelli*	48.55 ± 3.90	2.01 ± 0.08	3.30 ± 0.21	317.82 ± 25.12
*Phodopus sungorus*	46.82 ± 1.25	0.94 ± 0.06	1.58 ± 0.13	160.76 ± 27.07
*Phodopus roborovskii*	25.72 ± 1.15	1.06 ± 0.04	2.82 ± 0.12	175.64 ± 26.59

**Table 3:** Sperm motility, acrosome integrity, viability and ATP content in five hamster species. Values correspond to mean and standard error (SE) for spermatozoa in the absence (control) or presence of penicillamine, hypotaurine and epinephrine (PHE) at the start of the incubation period (time zero).

Species	Variable	Control		PHE	
		Mean	SE	Mean	SE
*Cricetulus griseus*	Motility (%)	80.00	1.58	83.00	2.00
	Acrosome Integrity (%)	88.60	2.62	89.20	2.92
	Viability (%)	94.00	1.41	95.60	1.29
	ATP content (amol cell^−1^)	449.34	51.76	489.47	53.91
*Mesocricetus auratus*	Motility (%)	79.17	2.01	84.17	2.01
	Acrosome Integrity (%)	86.83	2.21	85.50	1.61
	Viability (%)	98.67	0.42	97.83	0.75
	ATP content (amol cell^−1^)	437.68	63.39	368.09	16.23
*Phodopus campbelli*	Motility (%)	88.00	1.22	84.00	2.45
	Acrosome Integrity (%)	88.60	1.69	86.86	1.09
	Viability (%)	99.40	0.40	99.40	0.40
	ATP content (amol cell^−1^)	339.83	20.64	379.83	24.23
*Phodopus sungorus*	Motility (%)	84.29	1.70	81.43	1.80
	Acrosome Integrity (%)	79.71	3.89	74.86	3.74
	Viability (%)	98.43	0.48	98.29	0.36
	ATP content (amol cell^−1^)	369.20	59.19	435.80	83.28
*Phodopus roborovskii*	Motility (%)	89.00	1.00	87.00	1.22
	Acrosome Integrity (%)	86.80	3.14	87.40	2.62
	Viability (%)	96.40	0.93	96.40	1.69
	ATP content (amol cell^−1^)	459.57	33.29	467.05	32.20

**Table 4:** Effect of penicillamine, hypotaurine, and epinephrine (PHE) on sperm motility, acrosome integrity, viability, and ATP content, in five hamster species. *F* and *p* values correspond to repeated measures ANOVA using time of incubation and treatment (control vs. PHE) as independent variables and sperm parameters as dependent variables. Significant differences between treatments and times of incubation (*p* < 0.05) are shown in boldface.

Species	Variable	Time		Treatment		Interaction	
		*F*	*p*	*F*	*p*	*F*	*p*
*Cricetulus griseus*	Motility (%)	34.27	**<0.001**	9.14	**0.005**	1.34	0.280
	Acrosome Integrity (%)	44.62	**<0.001**	0.33	0.570	1.54	0.225
	Viability (%)	0.61	0.617	34.45	**<0.001**	2.53	0.078
	ATP content (amol cell^−1^)	35.39	**<0.001**	15.47	**0.001**	4.01	**0.007**
*Mesocricetus auratus*	Motility (%)	254.11	**<0.001**	110.40	**<0.001**	6.98	**0.001**
	Acrosome Integrity (%)	86.67	**<0.001**	6.66	**0.014**	0.24	0.870
	Viability (%)	2.10	0.118	6.04	**0.019**	2.00	0.132
	ATP content (amol cell^−1^)	120.43	**<0.001**	0.83	0.368	1.94	0.141
*Phodopus campbelli*	Motility (%)	16.81	**<0.001**	2.44	0.129	1.06	0.381
	Acrosome Integrity (%)	29.84	**<0.001**	5.17	0.085	0.22	0.880
	Viability (%)	1.69	0.192	1.13	0.298	1.97	0.142
	ATP content (amol cell^−1^)	43.92	**<0.001**	1.89	0.180	0.09	0.966
*Phodopus sungorus*	Motility (%)	13.17	**<0.001**	0.08	0.775	1.49	0.231
	Acrosome Integrity (%)	24.93	**<0.001**	1.54	0.261	0.92	0.442
	Viability (%)	0.42	0.743	0.98	0.327	0.55	0.648
	ATP content (amol cell^−1^)	37.52	**<0.001**	1.34	0.253	0.46	0.709
*Phodopus roborovskii*	Motility (%)	38.87	**<0.001**	1.06	0.312	0.44	0.727
	Acrosome Integrity (%)	18.66	**<0.001**	0.03	0.868	0.08	0.972
	Viability (%)	0.44	0.726	0.07	0.790	0.21	0.888
	ATP content (amol cell^−1^)	14.43	**<0.001**	1.46	0.237	0.12	0.950

**Table 5:** Sperm velocity and trajectory shape parameters in four hamster species. Values correspond to means and standard errors for spermatozoa in the absence (control) or with penicillamine, hypotaurine and epinephrine (PHE) at the start of the incubation period (time zero). VCL: curvilinear velocity. VSL: straight-line velocity. VAP: average path velocity. LIN: linearity. STR: straightness. WOB: wobble coefficient. ALH: amplitude of lateral head movement. BCF: beat-cross frequency.

Species	Variable	Control		PHE	
		Mean	SE	Mean	SE
*Mesocricetus auratus*	VCL (µm s^−1^)	114.71	2.40	123.45	3.99
	VSL (µm s^−1^)	54.66	2.33	56.76	2.27
	VAP (µm s^−1^)	76.64	2.02	82.22	2.26
	LIN (VSL/VCL)	0.47	0.02	0.46	0.02
	STR (VSL/VAP)	0.67	0.02	0.66	0.01
	WOB (VAP/VCL)	0.67	0.01	0.68	0.01
	ALH (µm)	5.17	0.11	5.67	0.22
	BCF (Hz)	6.32	0.18	6.27	0.18
*Phodopus campbelli*	VCL (µm s^−1^)	162.79	2.25	162.80	2.01
	VSL (µm s^−1^)	75.25	1.27	71.62	1.93
	VAP (µm s^−1^)	102.05	1.48	98.86	1.33
	LIN (VSL/VCL)	0.45	0.01	0.43	0.01
	STR (VSL/VAP)	0.70	0.01	0.69	0.01
	WOB (VAP/VCL)	0.62	0.01	0.61	0.01
	ALH (µm)	6.48	0.12	6.40	0.12
	BCF (Hz)	9.83	0.15	9.70	0.12
*Phodopus sungorus*	VCL (µm s^−1^)	152.02	2.24	157.70	2.29
	VSL (µm s^−1^)	68.81	2.36	69.10	2.19
	VAP (µm s^−1^)	92.19	1.91	92.81	2.13
	LIN (VSL/VCL)	0.44	0.01	0.42	0.01
	STR (VSL/VAP)	0.69	0.01	0.69	0.01
	WOB (VAP/VCL)	0.60	0.01	0.58	0.01
	ALH (µm)	6.34	0.11	6.56	0.11
	BCF (Hz)	8.70	0.34	9.21	0.28
*Phodopus roborovskii*	VCL (µm s^−1^)	152.85	2.91	146.54	1.01
	VSL (µm s^−1^)	62.13	1.85	56.36	2.42
	VAP (µm s^−1^)	87.26	1.68	80.80	1.50
	LIN (VSL/VCL)	0.40	0.01	0.38	0.02
	STR (VSL/VAP)	0.68	0.01	0.67	0.02
	WOB (VAP/VCL)	0.57	0.01	0.55	0.01
	ALH (µm)	6.96	0.12	7.14	0.11
	BCF (Hz)	8.40	0.35	7.53	0.48

**Table 6:** Effect of penicillamine, hypotaurine and epinephrine (PHE) on sperm velocity and trajectory shape parameters in four hamster species. *F* and *p* values correspond to repeated measures ANOVA using time of incubation and treatment (control vs. PHE) as independent variables, and sperm parameters as dependent variables. VCL: curvilinear velocity. VSL: straight-line velocity. VAP: average path velocity. LIN: linearity. STR: straightness. WOB: wobble coefficient. ALH: amplitude of lateral head movement. BCF: beat-cross frequency. VPC1: velocity principal component 1. TPC1: trajectory shape principal component 1. TPC2: trajectory shape principal component 2. Significant differences between treatments and times of incubation (*p* < 0.05) are shown in boldface.

Species	Variable	Time		Treatment		Interaction	
		*F*	*p*	*F*	*p*	*F*	*p*
*Mesocricetus auratus*	VCL (µm s^−1^)	28.78	**<0.001**	67.30	**<0.001**	3.54	**0.024**
	VSL (µm s^−1^)	14.62	**<0.001**	46.25	**<0.001**	4.43	**0.011**
	VAP (µm s^−1^)	29.51	**<0.001**	50.80	**<0.001**	3.26	**0.031**
	LIN	2.75	0.056	17.82	**<0.001**	2.83	0.074
	STR	1.04	0.384	19.68	**<0.001**	3.80	**0.017**
	WOB	15.50	**<0.001**	10.48	**0.002**	1.28	0.295
	ALH (µm)	0.68	0.573	26.39	**<0.001**	1.10	0.361
	BCF (Hz)	101.82	**<0.001**	93.48	**<0.001**	15.10	**<0.001**
	VPC1	25.98	**<0.001**	59.27	**<0.001**	4.09	**0.014**
	TPC1	5.12	**0.004**	9.61	**0.004**	2.45	0.077
	TPC2	7.08	**0.001**	73.25	**<0.001**	5.73	**0.003**
*Phodopus campbelli*	VCL (µm s^−1^)	12.90	**<0.001**	0.22	0.644	0.52	0.669
	VSL (µm s^−1^)	99.74	**<0.001**	4.53	**0.042**	0.09	0.964
	VAP (µm s^−1^)	83.46	**<0.001**	1.57	0.221	0.24	0.867
	LIN	61.32	**<0.001**	4.98	**0.034**	0.52	0.673
	STR	17.99	**<0.001**	1.51	0.230	0.29	0.832
	WOB	196.44	**<0.001**	14.20	**0.001**	1.11	0.363
	ALH (µm)	60.78	**<0.001**	7.96	**0.009**	2.29	0.100
	BCF (Hz)	43.82	**<0.001**	2.29	0.141	0.70	0.559
	VPC1	98.05	**<0.001**	2.15	0.154	0.15	0.931
	TPC1	70.65	**<0.001**	6.23	**0.019**	0.62	0.608
	TPC2	7.02	**0.001**	0.10	0.751	0.25	0.862
*Phodopus sungorus*	VCL (µm s^−1^)	8.19	**<0.001**	3.47	0.069	0.92	0.441
	VSL (µm s^−1^)	7.41	**<0.001**	2.46	0.124	0.63	0.603
	VAP (µm s^−1^)	9.70	**<0.001**	0.76	0.387	0.63	0.600
	LIN	2.75	0.055	6.27	**0.016**	0.32	0.814
	STR	1.88	0.148	2.75	0.105	0.59	0.626
	WOB	2.95	**0.043**	6.38	**0.015**	0.27	0.846
	ALH (µm)	1.02	0.394	5.36	0.060	0.09	0.966
	BCF (Hz)	1.85	0.153	0.02	0.899	1.11	0.355
	VPC1	9.79	**<0.001**	0.49	0.488	0.81	0.497
	TPC1	2.24	0.098	3.55	0.108	0.26	0.854
	TPC2	5.19	**0.004**	3.15	0.083	1.34	0.274
*Phodopus roborovskii*	VCL (µm s^−1^)	17.20	**<0.001**	4.75	**0.038**	0.22	0.879
	VSL (µm s^−1^)	2.61	0.071	8	**0.009**	0.60	0.620
	VAP (µm s^−1^)	0.89	**0.460**	9.34	**0.005**	0.42	0.739
	LIN	21.86	**<0.001**	2.99	0.095	0.29	0.833
	STR	9.20	**<0.001**	0.15	0.702	0.28	0.839
	WOB	20.12	**<0.001**	4.99	**0.035**	0.22	0.884
	ALH (µm)	30.78	**<0.001**	0.48	0.493	0.17	0.914
	BCF (Hz)	4.76	**0.008**	2.81	0.105	1.82	0.166
	VPC1	0.86	0.426	8.65	**0.006**	0.49	0.694
	TPC1	22.57	**<0.001**	2.33	0.138	0.27	0.843
	TPC2	11.21	**<0.001**	0.56	0.461	0.65	0.588

## Data Availability

Data are included in the article.

## Supplementary Materials

The following are available online at https://www.mdpi.com/article/10.3390/biology11040526/s1, Figure S1. Changes in sperm viability in five hamster species during incubation without or with penicillamine, hypotaurine and epinephrine (PHE). (A) *Cricetulus griseus*. (B) *Mesocricetus auratus*. (C) *Phodopus campbelli*. (D) *P. sungorus*. (E) *P. roborovskii*. Spermatozoa were collected in medium with (black squares) or without PHE (white squares) and incubated at 37 °C under air for up to 4 h. Values are means ± standard errors. Different letters between times of incubation for the same treatment indicate statistically significant differences (*p* < 0.05) in a DGC post hoc test. Asterisks indicate statistical differences (*p* < 0.05) between treatments for the same time in a DGC post-hoc test.
