# Supplementary material for: Effect of Motility Factors D-Penicillamine, Hypotaurine and Epinephrine on the Performance of Spermatozoa from Five Hamster Species
Source: Biology (Basel). 2022 Mar 30;11(4):526. doi: 10.3390/biology11040526 (PMC9032960; doi:10.3390/biology11040526)
Supplement: Supplementary file 1 [file biology-11-00526-s001.zip › biology-1603700-supplementary.pdf]

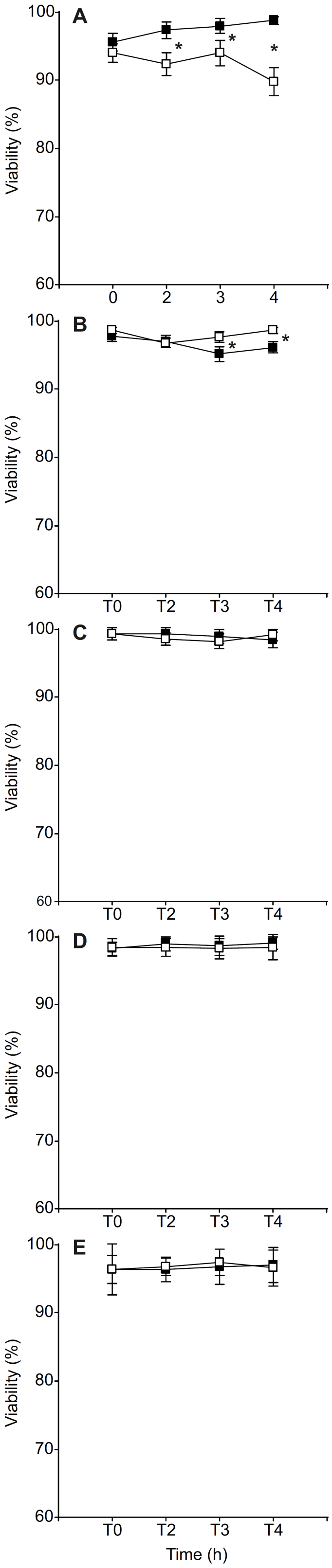

Figure S1. Changes in sperm viability in five hamster species during incubation without or with penicillamine, hypotaurine and epinephrine (PHE). A) *Cricetulus griseus*. B) *Mesocricetus auratus*. C) *Phodopus campbelli*. D) *P. sungorus*. E) *P. roborovskii*. Spermatozoa were collected in medium with (black squares) or without PHE (white squares) and incubated at 37ºC under air for up to 4 hours. Values are means ± standard errors. Different letters between times of incubation for the same treatment indicate statistically significant differences ($p < 0.05$) in a DGC *post-hoc* test. Asterisks indicate statistical differences ($p < 0.05$) between treatments for the same time in a DGC *post-hoc* test.
